# Channeling: A Non-pathological Possession and Dissociative Identity Experience or Something Else?

**DOI:** 10.1007/s11013-021-09730-9

**Published:** 2021-07-01

**Authors:** Luciano Pederzoli, Patrizio Tressoldi, Helané Wahbeh

**Affiliations:** 1 EvanLab, Florence, Italy; 2 Science of Consciousness Research Group, Studium Patavinum, Università di Padova, Padova, Italy; 3 Institute of Noetic Sciences, Petaluma, CA USA

**Keywords:** Channeling, Dissociative Identity Disorder, Non-ordinary mental experiences, Dissociative trance-possession disorders

## Abstract

Channeling experiences are often compared with Dissociative Trance/Possession Disorders and Dissociative Identity Disorders and more recent diagnostic criteria presented in the DSM 5 and ICD-11. From this comparison, it emerges quite clearly that, for most cases, channeling can either be considered an exceptional non-ordinary mental experience or a non-pathological Dissociative Trance/Possession experience. If this characterization is valid, the next step is to understand the origin of channeling experiences. Are they an expression of channeler’s unconscious or voluntary mental mechanisms, or real connections with “other discarnate entities”? Given their peculiar characteristics, channeling experiences offer a unique opportunity for a scientific investigation and in particular, the origin of the information received by the channelers.

## Introduction

### Channeling

Trance channeling has been defined by Klimo (1987:2) as “the communication of information to or through a physically embodied human being from a source that is said to exist on some other level or dimension of reality than the physical as we know it, and that is not from the normal mind (or self) of the channel.”

These experiences are common in many cultures, and their frequency varies according to their acceptance as a normal non-psychopathological expression (Cardeña et al. 2009; Luhrmann et al. 2001).

In recent years, a renewed interest has arisen in the scientific investigation of channeling within Western cultures that usually consider these experiences anomalous, exceptional, and probably expressions of mental disorders (Anastasia et al. 2020; Pederzoli et al. 2020; Stolovy, Lev-Wiesel, and Eisikovits 2015; Wahbeh et al. 2018b, 2019). This increase in research into channeling experiences is driven in part by the acknowledged commonality of the experience world-wide (Bourguignon 1976; Hunter and Luke 2014; Wahbeh, Radin, et al. 2018b; Wahbeh and Radin 2018).

But are channeling experiences different from dissociative trance/possession disorders (DTPD) as described by the updated international classification of mental disorders presented in the DSM-5 (American Psychiatric Association 2013) and ICD-11 (World Health Organization 2020)?

### Dissociative Trance/Possession Disorders

DSM-5 classifies dissociative trance as a Dissociative Identity Disorder ‘not otherwise specified’ (see Dissociative Identity Disorder paragraph).

ICD–11 description is the follow:

- “Possession trance disorder is characterised by trance states in which there is:
  - a marked alteration in the individual’s state of consciousness and
  - the individual’s customary sense of personal identity is replaced by an external ‘possessing’ identity and in which the individual’s behaviours or movements are experienced as being controlled by the possessing agent.
  - Possession trance episodes are recurrent or, if the diagnosis is based on a single episode, the episode has lasted for at least several days.
  - The possession trance state is involuntary and unwanted and is not accepted as a part of a collective cultural or religious practice.
  - The symptoms do not occur exclusively during another dissociative disorder and are not better explained by another mental, behavioural or neurodevelopmental disorder.
  - The symptoms are not due to the direct effects of a substance or medication on the central nervous system, including withdrawal effects, exhaustion, or to hypnagogic or hypnopompic states, and are not due to a disease of the nervous system or a sleep-wake disorder.
  - The symptoms result in significant distress or significant impairment in personal, family, social, educational, occupational or other important areas of functioning.”

### Criteria for Dissociative Identity Disorder in the DSM-5 (Tracy 2015)

The Dissociative Identity Disorder (DID) replaced the Multiple Personality Disorder diagnosis in the DSM-5. Despite ongoing debate about how to revise the criteria (e.g., Spiegel et al. 2013), the agreed criteria are:

1. Two or more distinct identities or personality states are present, each with its own relatively enduring pattern of perceiving, relating to and thinking about the environment and self.
  According to the DSM-5, personality states may be seen as an “experience of possession.” These states “involve(s) a marked discontinuity in sense of self and sense of agency, accompanied by related alterations in affect, behavior, consciousness, memory, perception, cognition, and/or sensory-motor functioning. These signs and symptoms may be observed by others or reported by the individual.”
  One important change from the fourth to the fifth edition of the DSM is that individuals may now report their perception of personality shifts rather than limiting diagnosis to shifts that others must report.

The second criterion is:

2. Amnesia must occur, defined as gaps in the recall of everyday events, important personal information and/or traumatic events. (Dissociative Amnesia: Deeply Buried Memories). This criteria for DID newly recognize that amnesia does not just occur for traumatic events but, rather, everyday events, too.
3. The person must be distressed by the disorder or have trouble functioning in one or more major life areas because of the disorder. This criterion is shared among all serious mental illness diagnoses as a diagnosis is not appropriate where the symptoms do not create distress and/or trouble functioning.
4. The disturbance is not part of normal cultural or religious practices. This DID criterion is to eliminate diagnosis in cultures or situations where multiplicity is appropriate. An example of this is in children where an imaginary friend is not necessarily indicative of mental illness.
5. The symptoms are not due to the direct physiological effects of a substance (such as blackouts or chaotic behavior during alcohol intoxication) or a general medical condition (such as complex partial seizures). This characteristic of Dissociative Identity Disorder is important as substance abuse or another medical condition is more appropriate to diagnose, when present, than DID”.

### Criteria for DID in the ICD -11 (WHO 2020)

- disruption of identity in which there are two or more distinct personality states (dissociative identities) associated with marked discontinuities in the sense of self and agency. Each personality state includes its own pattern of experiencing, perceiving, conceiving, and relating to self, the body, and the environment. At least two distinct personality states recurrently take executive control of the individual’s consciousness and functioning in interacting with others or with the environment, such as in the performance of specific aspects of daily life such as parenting, or work, or in response to specific situations (e.g., those that are perceived as threatening).
- changes in personality state are accompanied by related alterations in sensation, perception, affect, cognition, memory, motor control, and behaviour.
- there are typically episodes of amnesia, which may be severe.
- the symptoms are not better explained by another mental, behavioural or neurodevelopmental disorder and are not due to the direct effects of a substance or medication on the central nervous system, including withdrawal effects, and are not due to a disease of the nervous system or a sleep-wake disorder.
- the symptoms result in significant impairment in personal, family, social, educational, occupational or other important areas of functioning.

There are several ways in which channeling experiences differ from DID and DTPD. For example, when symptoms are measured with screening questionnaires, channelers do have higher symptom levels than controls. However, the symptom levels do not reach pathological levels (Castillo 2003; Alexander Moreira-Almeida and Cardeña 2011; Negro Jr. et al. 2002; Roxburgh and Roe 2011; Seligman 2005; Seligman and Kirmayer 2008; Stolovy, Lev-Wiesel, and Witztum 2015; Wahbeh and Butzer 2020).

Also, the amnestic criterium is rarely met. Most channelers remember their channeling experiences and do not have amnestic episodes in their daily lives (Negro, Palladino-Negro, and Louzã 2002; Wahbeh et al. 2019; Wahbeh and Butzer 2020).

The functional disability present in mental illness is also not found in channelers. Most people who have channeling experiences are well-adjusted, high-function individuals. Multiple studies have demonstrated these results on psychological well-being and distress, overall mental health, and social adjustment assessments (Negro, Palladino-Negro, and Louzã 2002; Moreira-Almeida and Cardeña 2011; Moreira-Almeida, Neto, and Cardeña 2008; Roxburgh and Roe 2011; Stolovy, Lev-Wiesel, and Witztum 2015; Moreira-Almeida, Neto, and Greyson 2007; Moreira-Almeida and Koss-Chioino 2009). In fact, one study found that mediums who experience being fully possessed by another entity had better social adjustment scores and fewer psychiatric symptoms than controls (Moreira-Almeida and Cardeña 2011).

Taking it one step further, not only are channelers well-adjusted and high-functioning, but they also express receiving positive benefit from their channeling experiences with the experiences being described as beneficial and even inspirational (Negro, Palladino-Negro, and Louzã 2002; Moreira-Almeida and Cardeña 2011; Wahbeh, Carpenter, and Radin 2018a; Wahbeh et al. 2019; Wahbeh and Butzer 2020).

Channelers believe their healing skills and providing information to their clients are valuable and serve a therapeutic function for them and their clients (Moreira-Almeida and Cardeña 2011; Roxburgh and Roe 2011; Emmons and Emmons 2003).

In some cultures, being a channeler provides practical gains such as increased community status, power and respect, and even livelihood. Channeling can also allow the channeler to view their traumatic life experiences with a different lens, stepping into the wounded healer archetype and perceiving their lifetime difficulties as preparation for their role as channelers (Seligman 2005).

Tu summarize, several consistent findings emerge from these investigations. The first is that channelers believe that their bodies are being used by “other identities” or “external non-physical beings” who communicate through them. These “other identities” are identified with multiple names and levels, from deceased humans to beings associated with specific religious traditions, e.g., angels, devas, etc. The communications’ content also have similar themes ranging from personal messages and guidance for the listeners’ spiritual and personal growth, to responses to scientific questions. Most relevant to this paper’s topic is the finding that most channelers do not reach thresholds for mental illness. Finally, and most importantly, most channelers find the experiences meaningful, beneficial, and positively impacting on their lives.

Other factors differ from mental illness such as switching to, locus of control, contact duration, awareness, number and psychopathology. In Table 1, we compared channeling characteristics with those of DID and DTPD.

**Table 1:** Channeling, DID and DTPD comparison

“Other identities”	Channeling	DTPD	DID
Switching to	**Controlled**	Uncontrolled	Uncontrolled
Locus of	External	External	Internal
Contact duration	**Controlled**	Variable	Uncontrolled
Awareness	**Variable**	Reduced or abolished	Reduced
Number	One at time	One at time	At least two
Psychopathology			
Dissociative Identity Disorders	**Rare**	Always	Always
Functioning impairment	**Rare or mild**	Always	Always

* DID* Dissociative Identity Disorder, *DTPD* Dissociative trance/possession disorders

Bold: characteristics which differentiate channeling from DID and DTPD

## Discussion

Channeling experiences are clearly distinct from DID and DTPD. Hence, channeling can either be considered an exceptional non-ordinary mental experience or a non-pathological dissociative trance/possession experience.

If this characterization is valid, the next step is to attempt to understand the origin of this experience. For example, some hypotheses for DID and DTPD state that they are expressions of unconscious or voluntary mental mechanisms activated for filtering out the memory of past or present physical or emotional trauma (Spiegel et al. 2013) or of cognitive characteristics like memory errors, cognitive failures, problems in attentional control and difficulties in distinguishing fantasy from reality (Lynn et al. 2012). Are these hypotheses valid for the origin of channeling experiences?

Are the purported “other identities”, different expressions of the channeler personality? If so, why and how does the channeler create such identities? Or are “other identities” real independent identities living in another or other worlds? According to the channelers’ personal reports, the latter is true. The same interpretation is shared by many cultures and religions, as well documented by Luhrmann et al. (2021).

However, how is it possible to tackle this hypothesis from a scientific point of view? Despite some work to test the veracity of channeled information from deceased persons using rigorous methods such as triple-blinded study designs, there is no definitive evidence of survival of human consciousness or the existence of non-physical entities (Beischel et al. 2015; Beischel and Schwartz 2007; Delorme et al. 2013, 2018; Sarraf, Woodley, and Tressoldi 2020).

If channelers are truly channeling “other identities,” how can we disentangle this from the possibility that “other identities” are expressions of the channelers’ different personalities? Here we list some means we think useful for responding to this question:

- Is it possible to dialogue with the “other identities”? If so, the fact that this dialogue takes place between or among different channelers’ personalities seems quite odd or at least, more complex to explain. Preliminary findings from Pederzoli et al. (2020) indeed found just that with selected participants who had channeling experiences induced by using hypnotic suggestions. These participants channeled different “other identities” who responded to multiple questions posed by the hypnotist from a variety of disciplines such as physics, biology, and psychology.
- Is it possible to ask the channelers identical pool of questions, which they respond to in a normal state of consciousness and then in a channeling state, and then compare the responses obtained in these two different states of awareness? If the responses and manner of expression are different, which is the more plausible interpretation of their origin?
- Is it possible to ask the same “other identities” to respond to specific questions using different channelers? If so, how could different channelers manifest identical “other identities” as expressions of their personality?
- Channeling “other identities,” is it possible to observe special skills (i.e., artistic talent or specialized knowledge), unknown to the channeler? This question has been explored in multiple cases ranging from extraordinary artistic production, literary works, and musical composition, all of which do not align with the channelers’ abilities or education (Braude 2003:133–134, 166–169; Hageman, Krippner, and Wickramasekera 2011; Hastings 1991:165; Maraldi 2014; Maraldi and Fernandes 2020).

While answering these questions may not give definitive answers, it will indeed allow us to elucidate the original of channeling experiences. Given its peculiar non-pathological status and commonality, channeling experiences offer a unique opportunity to scientifically investigate their characteristics without harming the channelers and, in particular, the origin of the information they receive.
